# Cerebrospinal Fluid Extracellular Vesicles Undergo Age Dependent Declines and Contain Known and Novel Non-coding RNAs

**DOI:** 10.1371/journal.pone.0113116

**Published:** 2014-11-24

**Authors:** Ashlee Tietje, Kourtney N. Maron, Yanzhang Wei, David M. Feliciano

**Affiliations:** Department of Biological Sciences, Clemson University, Clemson, South Carolina, United States of America; Colorado State University, United States of America

## Abstract

Brain development requires precise orchestration of cellular events through the coordinate exchange of information between distally located cells. One mechanism by which intercellular communication is achieved is through the transfer of extracellular vesicles (EVs). Exosomes are EVs that carry lipids, nucleic acids, and proteins and are detectable in most biological fluids including cerebrospinal fluid (CSF). Here we report that CSF EV concentrations undergo age dependent fluctuations. We characterized EV RNA content by next generation small RNA sequencing and miRNA microarray analysis and identified a temporal shift in CSF EV content. CSF EVs encapsulated miRNAs that contain a conserved hnRNPA2/B1 recognition sequence. We found that hnRNPA2/B1-containing EVs were produced by choroid plexus epithelial cells and that hnRNPA2/B1 containing EVs decreased with age. These results provide insight into EV exchange of miRNAs within the central nervous system and a framework to understand how changes in EVs may have an important impact on brain development.

## Introduction

Intercellular communication *via* EVs is a common feature of developing tissues and is often achieved through coordinated release and uptake of EV constituents [1]. EVs help to establish morphogenetic gradients (argosomes), facilitate tissue innervation (exosomes), generate stem cell niches (microvesicles, vesicles, and exosomes), and can facilitate a range of pathological processes [2]–[9]. Exosomes are nanometer-sized EVs with a phospholipid bilayer membrane, which encapsulates proteins and nucleic acids [1], [5], [10]–[12]. Although microvesicles can bud from the cell membrane, exosomes are distinct and are thought to originate upon membrane invagination of multivesicular bodies (MVBs) *via* an ESCRT-dependent pathway and are released when MVBs fuse to the cell membrane [13]. Exosomes bind to heparin sulfate proteoglycans on recipient cells, fuse with the plasma membrane, and subsequently transfer their content [14]. Since EVs carry proteins, mRNA, and microRNA (miRNA) to target cells, they are unique in that they have the potential for multilayer regulation of cellular physiology which contrasts the top-down signaling coordinated by classical ligand activated receptors. Thus, EVs represent a mechanism for coordinating complex physiological processes and may play an important role in maturation of the CNS.

EVs are detectable in most biological fluids including CSF [5], [15]–[21]. CSF EVs encapsulate miRNA during embryonic development in rodents and humans and may promote neural stem cell (NSC) amplification [5]. However, the role that EVs and, in particular, exosomes may play within the CNS may depend on the source from which they originate and the target cell. For example, CSF exosomes from Alzheimer's patients can induce astrocytic cell death [16]. Alternatively, the choroid plexus, which produces CSF, may transfer EVs thereby shuttling folate to the parenchyma [22]. Nevertheless, the quantity, biochemical characteristics, and content of CSF EVs remain undefined.

Here, we describe that CSF EVs are subject to age dependent fluctuations. These EVs are abundant during perinatal development but precipitously decline throughout life. Next generation sequencing of EV content identified novel small RNAs as well as those previously described. The miRNA content within the CSF EVs was also dependent on age and these miRNAs contain a previously described sorting consensus sequence recognized by hnRNPs [23]. In agreement, our data show that the CSF EVs contain hnRNPA2/B1and these EVs were subject to age dependent declines. Additionally, the choroid plexus produces hnRNPA2/B1 containing EVs. Taken together, these results highlight that CSF EVs and their contents undergo temporal fluctuations during brain development and that these changes may be of physiological significance.

## Material and Methods

### CSF tracking analysis and EV isolation

Human CSF was stored at −80°C. Samples were thawed on ice and centrifuged at 2,000×g for 15 minutes. 800 µL samples were shipped on dry ice to the Nanomedicine Characterization Core facility in the Center for Nanotechnology in Drug Delivery at UNC at Chapel Hill. Samples were handled in a laminar flow hood, sequentially thawed at room temperature, and then diluted 20–500 fold based on an initial run with Phosphate Buffered Saline (PBS) (10 mM salt) and if required, additionally diluted 100 fold. Samples were then loaded onto a pre-cleaned and pre-warmed (30 minutes) Nanosight NS 500 nanoparticle characterization system (NanoSight, NanoSight Ltd, UK) equipped with green laser (532 nm) illumination. Mean size and particle concentration values were calculated by the nanoparticle tracking software which allows analysis of video images for calculation of the diffusion coefficient, sphere equivalent, mean-square displacement, and hydrodynamic radius of particles by using the Strokes-Einstein equation. The Nanosight NS 500 was calibrated with 100 nm polystyrene latex microsphere standards (Nanosight Ltd, UK) and all experiments were performed at a stabilized temperature of 23.3°C. Three to twenty-eight runs were performed for each sample. For isolation of vesicles and vesicle contents, where specified, pre-cleared CSF supernatant (or cell culture media) was placed onto a GE Healthcare S400-HR sephacryl column and centrifuged for 2 minutes at 735×g. EVs were precipitated using total exosome isolation reagent (from cell culture media), (other fluids isolation), or Exoquick tissue culture media exosome precipitation solution, (Life Technologies and Systems Biosciences). Samples were placed on a rotisserie overnight at 4°C. Samples were subsequently centrifuged at 2000×g at 4°C for 15–30 minutes. Supernatant was removed and cell pellets were used for protein or RNA isolations.

### EV RNA isolation

RNA isolations were performed as previously described [5]. Briefly, to isolate total RNA, Trizol was added to EV pellets derived from approximately and vortexed for 15 seconds. Samples were incubated for 5 minutes at room temperature, chloroform added, manually agitated for 15 seconds, incubated for an additional 5 minutes at room temperature, and centrifuged for 15 minutes at 12,000×g at 4°C. The aqueous phase was transferred to a fresh tube and isopropyl alcohol added to precipitate RNA. Samples were incubated overnight at −20°C then centrifuged for 10 minutes at 12,000×g at 4°C. Supernatant was discarded and pellets were washed with 70% ethanol, vortexed, and centrifuged for 5 minutes at 12,000×g at 4°C. The previous step was repeated and samples dried and rehydrated in RNase/DNase free DEPC treated water.

### Electron Microscopy

Electron microscopy was performed by the Yale imaging core facility as we recently described [5]. Briefly, EVs were resuspended in 4% wt/vol paraformaldehyde in phosphate buffered solution (pH 7.4) and embedded for 20 minutes at room temperature in a formvar-carbon-coated grid. The embedded vesicles were washed in phosphate buffered saline (PBS), fixed in 1% gluteraldehyde for 5 minutes, and stained with saturated aqueous uranyl oxalate. Samples were subsequently embedded in 0.4% wt/vol uranyl acetate and 1.8% wt/vol methylcellulose on ice for 10 minutes. Samples were dried at room temperature prior to visualization with a Carl Zeiss 910 electron microscope (Carl Zeiss Microscopy, Thornwood, NY).

### Ethics Statement

Approval for the acquisition of CSF samples was sought through the Clemson University Office of Research Compliance (ORC) and Institutional Review Board (IRB). The Clemson University ORC determined that the project does not involve human subjects as defined in the federal regulations governing the protection of human subjects in research and is, therefore, not subject to IRB review. This project did not involve either “intervention or interaction” with living individuals, or the collection or use of “identifiable private information” about living individuals. Samples were not collected specifically for the described research and no individually identifying information was acquired for these studies. Nevertheless, samples were obtained with consent that samples would be used for manufacturing or research purposes. Samples were handled in accordance with ethical guidelines and regulations for the research use of human tissue set forth by the NIH. All samples were de-identified according to HIPAA standards. Patients were categorized by age. Eleven patients were in categorized in group A and ranged from 11 days to 2 years of age. Patients in group B ranged in age between 10 years of age to 15 years and included three patients. There were nine patients in group C and ranged in age from 70 years to 100 years.

### miRNA microarrays

Pooled EV RNA derived from CSF samples from two patients falling within the same age category were processed by the Clemson University Genomics Institute (N = 3 microarrays for each condition, n = 1–2 patients for each microarray). Samples were subjected to analysis on an Agilent Technologies Bioanalyzer. 500 ng of RNA was subjected to Poly A tail labeling and ligation with the FlashTag Biotin Labeling kit (Affymetrix, Santa Clara, CA) and subjected to ELOSA QC analysis. Samples were hybridized to Affymetrix Genechip miRNA 3.0 arrays (Affymetrix, Santa Clara, CA) for 16 hours at 45°C, washed, stained with an Affymetrix fluidics station 450s, and scanned using a 16-bit Genechip scanner 3000 7G platform (Solid State 532 nm Diode-pumped Frequency Doubled neodymium-doped yttrium aluminum garnet green laser). Samples were analyzed using the Affymetrix GeneChip Command Console software and TM4 microarray software suite using hierarchical clustering analysis. Alternatively, sequence analysis of miRNA was performed using Clustal Omega [24]. Data is deposited as a MIAME compliant study to the NCBI Gene Expression Omnibus as GEO accession number GSE57713.

### Cell Culture

Human choroid plexus epithelial cells (ScienCell Research Laboratories) were routinely cultured in 20 mL epithelial cell media (EpiCM, ScienCell Research Laboratories) supplemented with 5% Fetal Bovine Serum, and 100 units/mL penicillin, and 100 µg/mL streptomycin in T-75 flasks pre-coated with Poly-L-lysine. Cells were plated at a density of 500,000 cells per flask and media replaced every 3 days. Cells were maintained at 37°C in a Revco Ultima CO_2_ incubator with 5% CO_2_, 95%. For routine passaging of cells, media was removed once cultures reached 70% confluence; cells were rinsed with PBS, and incubated with Trypsin/EDTA in PBS (1∶1) for three minutes. Trypsin/EDTA solution was removed, and placed into a 50 mL tube containing 5 mL FBS. Flasks were incubated for an additional 1 minute followed by the addition of 5 mL of PBS, 10% FBS, followed by trituration and cell suspension transferred to the 50 mL tube containing Trypsin/EDTA and FBS. Flasks were rinsed with an additional 5 mL PBS, 10% FBS which was subsequently added to the 50 mL tube. Cells were centrifuged at 1000 rpm for 5 minutes, supernatant was decanted and cells were suspended in complete media. Where indicated cells were suspended in the absence of serum in Neurobasal A, 1 mM sodium pyruvate, B27, N2, 100 pg/mL hEGF, 100 units/mL penicillin, and 100 µg/mL streptomycin (serum free media). Two to three days after passaging into cell free media, serum free media was replaced with fresh serum free media. Media was then collected two to three days later, EVs isolated, and western blotting performed.

### Next generation small RNA sequence analysis

Pooled EVs derived from CSF samples taken from six patients less than 2 years of age were subject to RNA isolations. Two pooled samples were generated, one containing samples from two patients and the other containing samples from four patients. Briefly, total RNA was isolated by the Trizol method. Small RNA was prepared according to manufacturer's protocol (Illumina small RNA preparation kit). Samples were subjected to Paired-end 90–100 nucleotide reads on an Illumina HiSeq2000.

### CSF Acquisition

CSF was isolated via lumbar puncture and maintained as individual collections during and after the procedure, up until the time they are picked up by couriers to be delivered to one of several sites for processing for research purposes. The specimens were released for “disposal” once they were determined to be free of any know pathology(ies) by the current test modalities available to clinicians. Samples were refrigerated for a period of time until they are “released” by the pathology department as being no longer necessary to retain.

Findings from immunoblots were also confirmed with additional samples isolated by lumbar or ventricular puncture. Human tissue was obtained from University of Maryland Brain and Tissue Bank which is a Brain and Tissue Repository of the NIH NeuroBioBank. Human tissue was also acquired from the Carroll A. Campbell, Jr. Neuropathology Lab at the Medical University of South Carolina. Samples were derived from patients less than 2 years that had grossly unremarkable neuropathological findings. Patients greater than 70 years had cancer or heart disease with no clinical history of dementia, but one patient contained neurofibrillary tangles.

### Immunoblotting

Equal volumes of human CSF were subjected to EV isolation. EVs were incubated with equal volumes of Trizol or lysed in equal volumes of RIPA buffer. Upon removal of the upper aqueous layer, proteins were subjected to methanol-chloroform extraction as described previously and rehydrated in RIPA buffer. Equal volumes of Laemmli buffer were then added to samples and immunoblotting was performed as previously described [5]. Briefly, EVs from equal volumes of CSF were resolved by standard electrophoresis conditions on 10% polyacrylamide precast mini-Protean TGX gels and transferred to polyvinylidene difluoride membranes. Membranes were rinsed in TBST (Tris-buffered saline, 0.1% Tween 20) for 5 minutes at room temperature and subsequently blocked in 5% w/v nonfat milk in TBST for 1 hour at room temperature. Following three rinses (each of 5 minutes) at room temperature in TBST, samples were incubated for 1 hour at room temperature or overnight at 4°C with the following antibodies: ALIX (Cell Signaling Technology, Danvers, MA, 1∶000) and hnRNPA2/B1 (2A2) (Cell Signaling Technology, Danvers, MA, 1∶1000). Following an additional three rinses each of 5 minutes in TBST, samples were incubated for 1 hour at room temperature with donkey or goat anti-rabbit antibodies in blocking buffer and then subjected to four 15-minute washes in TBST and visualized by enhanced chemiluminescence. Films were scanned at tiff files and uploaded for densitometric analysis using Quantity One software (Bio-rad). All bands corresponding were outlined by the rectangle volume analysis tool. Densitometric values are represented as abundance relative to the average abundance for samples from patients less than two years of age.

### Statistics

Determination of statistical significance for immunoblots for EV markers was performed for each group by using Student's T-test. Statistical analysis for nanoparticle tracking analysis was performed using ANOVA (GraphPad Prism 6).

## Results

### Age dependent changes in CSF vesicles

To determine the concentration and to measure the size of EVs within CSF, nanoparticle tracking analysis was performed. Following calibration using 100 nm-diameter polystyrene latex microspheres, CSF samples were analyzed from patients in each of the following three categories: less than 2 years of age (Group A), 10–15 years (Group B), or 70–85 years (Group C). Approximately three to six readings from each patient were taken.

**Figure 1:**
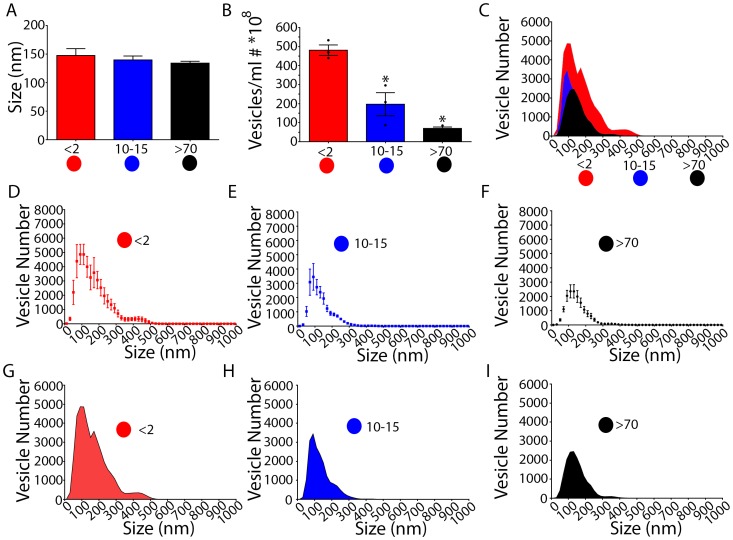
CSF EVs undergo temporal declines. A. CSF from patients ranging from 1 month to 85 years was collected and subjected to nanoparticle tracking analysis. The average size of EVs was unchanged. B. The average number of EVs was highest in patients less than 2 years and decreased by 10–15 years and 70 years. C. Overlay of size distribution of vesicles from patients less than 2 years, 10-15 years, and greater than 70 years. D–F. Size distribution averages with SEM depicted. G–I. Size distribution averages with area under the curve depicted. *: p<.01 Error Bars: SEM.

The average size of EVs in each group was determined (**Figure 1A**). There were no statistical differences in the average size of EVs among the three groups analyzed (A,B,C, mean ± SEM of 147.5±12.1, 139.8±6.90, 134.1±3.4 respectively, One way ANOVA, p = 0.4210). Next, the average number of EVs in each group was quantified. Group A patients had more total vesicles than either groups B or C (One way ANOVA on N = 3,3,3, means of 481.1±27.5 (A), 197.1±60.6 (B), 69.8±.58 (C), P = 0.0008) (**Figure 1B**). Compared to Group A, there was a 60% and 85% reduction in EVs for Group B and Group C, respectively.

We also examined EV sizes based upon frequency histogram analysis (**Figure 1C–I** **)**. The reduction in EV number was independent of size as seen with overlaid frequency histograms (**Figure 1C**). We found a continuum of vesicle sizes within the CSF at all ages with prominent populations being found between 50–200 nm (**Figure 1D–I**). Prominent peak populations were observed from 50–100 nm, 100–150 nm, and 150–200 nm (**Figure 1D–I**).

**Figure 2:**
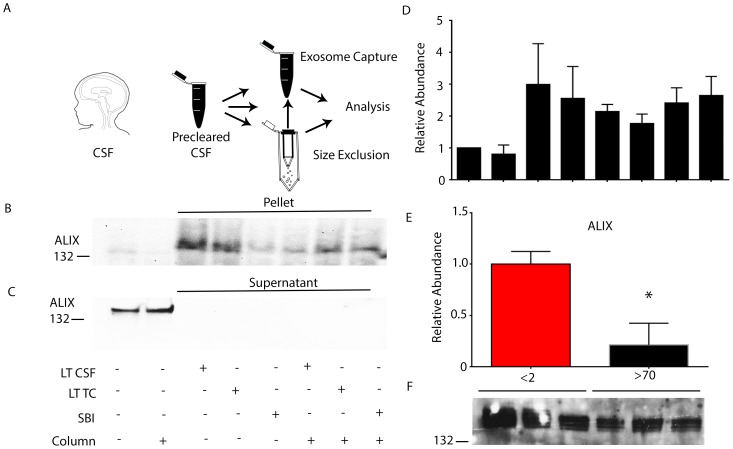
CSF EVs undergo temporal declines. A. Schematic representation of EV isolations. B. Representative immunoblot for ALIX following EV enrichment using size exclusion and then precipitation. C. Representative immunoblot of remaining ALIX after the depletion of EVs from CSF by size exclusion and precipitation. D. Quantification of ALIX immunoreactivity corresponding from left lane to right lane of B. E. Quantification of ALIX in CSF from patients less than 2 years and greater than 70 years. F. Corresponding immunoblots demonstrating age dependent decreases in ALIX. *: p<.01 Error Bars: SEM.

We hypothesized that a fraction of, CSF EVs may be exosomes based on size [1]. To test this hypothesis, we utilized several methodologies to isolate EVs from patient CSF (**Figure 2A**). First, we utilized size exclusion chromatography by filtering CSF over GE Healthcare S400-HR sephacryl columns. These columns extract all molecules up to 30 nm in size which is sufficient to remove LDL or HDL particles (approximately 20 nm). We also used Life Technologies Exosome Tissue Culture, Life Technologies Exosome Other Fluid, or System Biosciences Exosome Tissue Culture Isolation Reagent. Finally, we examined the combination of size exclusion chromatography with the aforementioned isolation reagents. Then western blots were performed on either supernatants (**Figure 2B**) or pellets using each methodology (**Figure 2C**). Size exclusion chromatography did not enrich samples for vesicle marker proteins compared to pure CSF fractions. However, exosome isolation reagents alone or following size exclusion chromatography greatly enriched samples for the exosome marker ALIX. In addition, we examined extracted vesicles for CD9 and CD133, a marker of large 600 nm vesicles derived from neural stem cells and CSF [6], [25]. CD133 was undetectable using our preparations but detectable in embryonic cortical tissue extracts (data not shown). However, we consistently detected CD9 (data not shown).

We next performed size exclusion chromatography followed by polymer extraction using SBI tissue culture isolation reagent on equal volumes of CSF from patients less than 2 years or greater than 70 years of age. EV proteins were isolated and analyzed by Western blot (**Figure 2D, 2E** **)**. Patients greater than 70 years had 79% less ALIX compared to patients less than 2 years of age (: 1.00±0.12 vs 70 years: 0.21±0.21 N = 6,6 patients P = 0.0092, Student's t-test) (**Figure 2D, 2E**).

### Identification of CSF EV miRNAs from patients under 2 years of age

**Figure 3:**
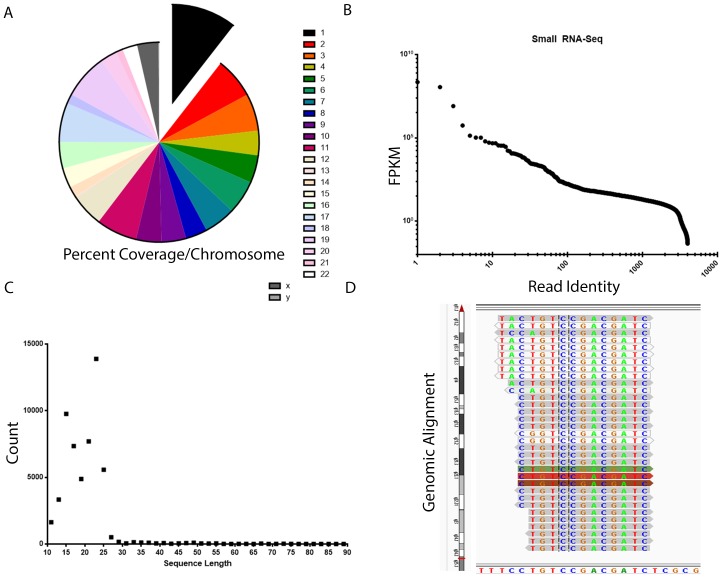
Next generation sequencing of CSF vesicle RNA content from patients less than 2 years old. A. Reads aligned to the human genome as a distribution across chromosomes. B. Rank sum distribution of mapped reads as a function of relative abundance (FPKM). C. Frequency distribution of reads in relation to read length. D. Alignment of a novel short RNA, CUFF .1222 to Hg19 chromosome 17 as visualized by integrated genomics viewer. The sequence of Hg19 is seen on the X axis. The chromosomal alignment is shown on the Y axis with red delineating the boundaries of the alignment.

We performed EV isolations on CSF from patients less than two years of age, followed by RNA extraction, and next generation small RNA sequencing. An average of 26,349,176 reads were generated, with an average of 4.23% of reads mapped per chromosome of Hg19, although deviations were found including for chromosome 1 for which 10.53% of reads were mapped (**Figure 3A**). Rank sum profile of reads indicated that EVs contain a small proportion of high abundance small RNAs mapping to 4,004 unique locations (**Figure 3B**). The majority of mapped reads were approximately 15–25 nucleotides in length characteristic of miRNAs (**Figure 3C**). Of the top 30 mapped reads which were responsible for, 19/30 mapped to known miRNAs including the MIR17–92 cluster, MIR30A and D, MIR10A and B, MIR22, MIR204, MIR375, MIR143, MIR99B, MIR320A, MIR27B, MIR140, MIR26A1 and A2, and MIR125B1 and MIR125B2 (**Table 1**). In addition several novel short RNAs, for example Cuff .1222 aligned to the genome (**Figure 3D**) and are predicted by DIANA - microT v3.0 to selectively repress specific target mRNAs by binding to the 3′ UTR of transcripts.

**Table 1:** Reads from next generation sequencing of vesicle RNA isolated from CSF (less than 2 years) and aligned to the human genome.

Gene ID	Gene Short Name	Locus
CUFF.385		chr10: 4298084–4298101
CUFF.1222		chr15: 78730469–78730484
CUFF.1519		chr17: 33478206–33478275
CUFF.2263		chr21: 9827404–9827458
MIR30A	MIR30A	chr6: 72113253–72113324
CUFF.1437	MIR22,MIR22HG	chr17: 1614797–1619566
MIR204	MIR204	chr9: 73424890–73425000
MIR375	MIR375	chr2: 219866366–219866430
CUFF.2853	MIR143,MIR143HG,MIR145	chr5: 148786439–148812399
MIR99B	MIR99B	chr19: 52195864–52195934
MIR30D	MIR30D	chr8: 135817118–135817188
MIR92B	MIR92B	chr1: 155164967–155165063
MIR10A	MIR10A	chr17: 46657199–46657309
MIR10B	MIR10B	chr2: 177015030–177015140
MIR320A	MIR320A	chr8: 22102474–22102556
MIR27B	MIR27B	chr9: 97847726–97847823
MIR140	MIR140	chr16: 69966983–69967083
CUFF.1451	GSG2	chr17: 3627196–3629992
MIR125B1	MIR125B1	chr11: 121970464–121970552
MIR125B2	MIR125B2	chr21: 17962556–17962645
CUFF.2266		chr21: 15136517–15136703
MIR92A2	MIR92A2	chrX: 133303567–133303642
MIR26A1	MIR26A1	chr3: 38010894–38010971
CUFF.1003	MIR17,MIR17HG,MIR18A,MIR19A,MIR19B1,MIR20A,MIR92A1	chr13: 92000073–92006829
CUFF.3276	MIR54802	chr8: 29953398–29953607
MIR26A2	MIR26A2	chr12: 58218391–58218475
CUFF.543		chr10: 135163541–135163793
CUFF.326	LGR6	chr1: 202163117–202288889
CUFF.1776		chr19: 11129458–11129782

### CSF vesicles contain small RNA

**Figure 4:**
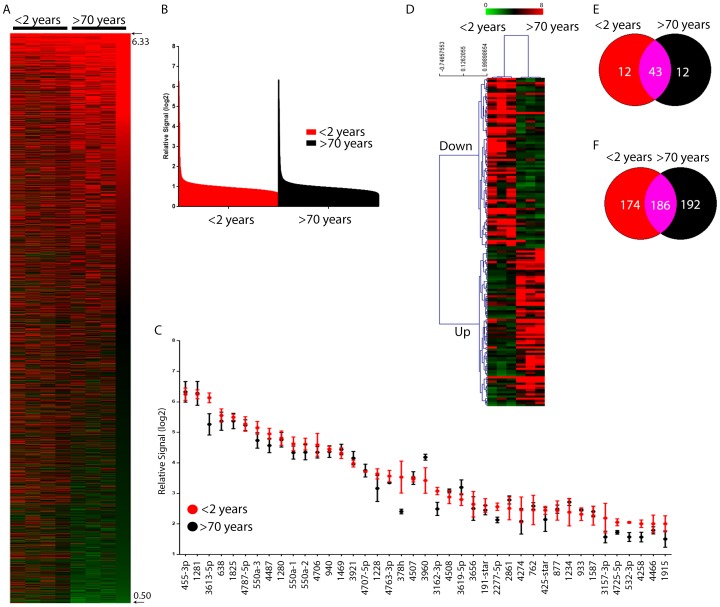
Temporal dynamics of CSF vesicle miRNAs. A. miRNA microarray heat-map of CSF vesicle content from patients less than 2 years or greater than 70 years where red is higher expression and green is lower expression. B. Rank expression of miRNAs based on microarray probe intensity. Red is microarray data from patients less than 2 years and black is from patients greater than 70 years. C. Log(_2_) signal of the most abundant miRNAs. D. Hierarchical clustering of miRNA microarrays at different ages. E. Venn diagram of high abundance or (F.) low abundance miRNAs at different ages.

We performed miRNA microarray analysis of CSF EV samples from patients less than 2 years (Group A) or greater than 70 years of age (Group C) (**Figure 4A**). Rank expression profiles including range were similar for both groups of patients (**Figure 4B**). Highly expressed miRNAs, expression levels were similar amongst both age groups (**Figure 4C**). Hierarchical clustering of miRNA and samples using TM4 software classified miRNAs into two categories (<2 years or >70 years) based on expression (**Figure 4D**). Of the differentially clustered miRNAs, 2.2% were significantly down-regulated and 2.34% were significantly up-regulated. Using an artificial cut-off of 0.90 log_2_ fold expression, approximately 42 miRNAs were uniquely expressed in vesicles from group A patients compared to 32 expressed in group C patients. More than 50 miRNAs were expressed log_2_ (2.0) fold above background in both conditions of which 43 were shared (**Figure 4E**). Lower expressed miRNA (log_2_ (1.30) to log_2_ (2.0) fold) had 48.3% and 50.8% unique miRNAs (percentage based on expression range) for patients <2 years and >70 years, respectively (**Figure 4F**).

### Choroid plexus epithelial cells (CPEs) generate EVs-

**Figure 5:**
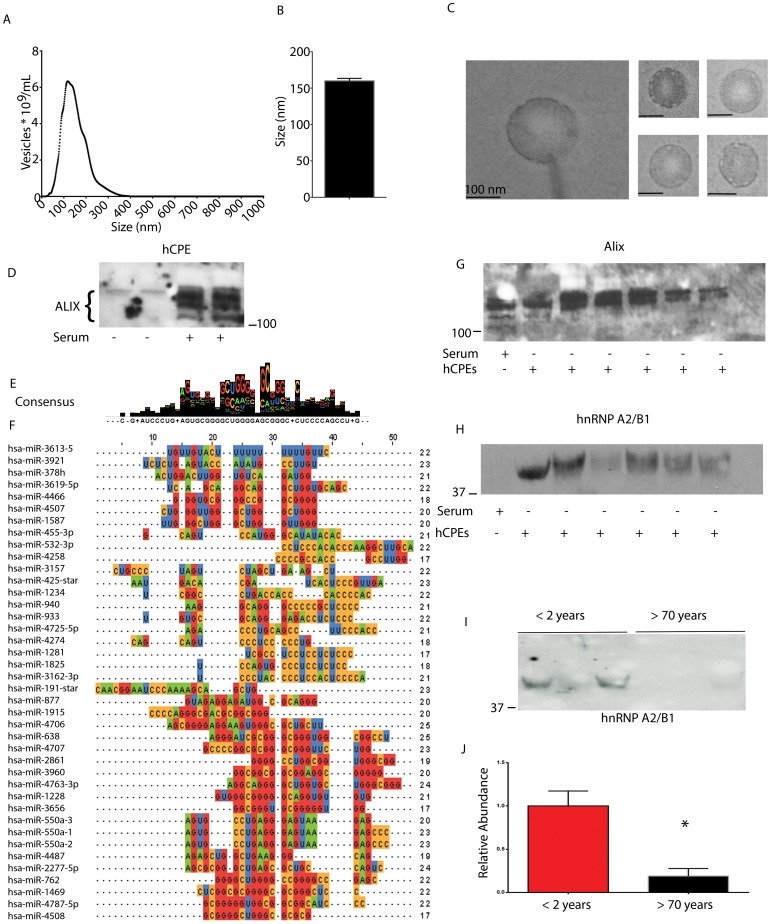
hnRNP A2/B1 is a choroid plexus and CSF vesicle protein. A. Nanoparticle tracking analysis of serum free media from human CPE cultures and size frequency distributions. Media contained 559.1 (x10^9^) EVs per mL (N = 3). B. The average size of an EV was 160.3 nm (N = 3). C. Transmission electron micrographs of CPE EVs. Scale bar: 100 nm. D. Immunoblot of the exosome protein ALIX from vesicles isolated from CPEs cultured in the presence or absence of serum. E. The consensus sequence contains features of a recently described exosome miRNA motif recognized by hnRNPA2/B1. F. Multiple sequence alignment of vesicle proteins indicates conservation of miRNA sequence. G. Immunoblot of CPE derived media in the absence of serum or media alone containing serum for ALIX H. Immunoblot of hnRNPA2/B1 from CPE derived media in the absence of serum or media alone containing serum for. I. Immunoblot of hnRNPA2/B1 from CSF EVs demonstrating an age dependent decrease. J. Quantification of immunoblots for hnRNPA2/B1. *: p = 0.0138 Error Bars: SEM.

Human CPEs were cultured to determine whether they might produce EVs. CPEs had perinuclear, small cytoplasmic and large vesicles (data not shown) as reported elsewhere [26]. CPEs were cultured in the absence of serum for two days, serum free media was replaced, and two-three days later media was collected. CPE conditioned media was subjected to NTA analysis. CPEs produced an average of 559.1 (x10^9^) EVs per mL (SEM ±102.3, N = 3 cultures, n = 9 readings) (**Figure 5A**). The average size of an EV was 160.3 nm (SEM ±3.416 nm, N = 3 cultures, n = 9 readings) (**Figure 5B**). To confirm our findings that detected particles were EVs, electron microscopy was performed. CPE EVs were isolated and subjected to transmission electron microscopy. Electron micrographs demonstrated the presence of 150 nm EVs (**Figure 5C**). Cells were cultured in serum free media and two to three days later the media was replaced with more serum free media. Two to three days after replacing serum free media, EVs were isolated from serum free media. Equal volumes of cell culture media were removed, pre-cleared, and subjected to vesicle isolation. Precipitated EVs were lysed and subjected to immunoblot analysis. ALIX was detected in EVs from conditioned media from both conditions (**Figure 5D**).

We hypothesized that the unique compendium of miRNA (4.5% of miRNAs) within vesicles might depend upon primary sequence determinants which dictate encapsulation. To identify primary sequence determinants, highly expressed miRNAs were subjected to multiple sequence alignment using Clustal Omega (**Figure 5F**). Sequences of mature miRNAs were derived from MiRBase. Multiple sequence alignment of CSF vesicle miRNA revealed a consensus sequence of GCUGGGGAGCGGG (**Figure 5E**). The consensus sequence contains a recently described miRNA motif of GGAG present in human T-lymphocyte exosome miRNAs that is recognized by hnRNPA2/B1 [23]. We therefore examined hnRNPA2/B1 levels and ALIX as a control in EVs from FCS or serum-free media collected from cultured CPEs (**Figure 5G, 5H**). We detected ALIX but did not detect hnRNPA2/B1 from FCS alone. However, hnRNPA2/B1 was readily detected in EVs derived from CPEs cultured in the absence of serum. The presence of hnRNPA2/B1 containing EVs from choroid plexus suggested the possibility that these vesicles may be present in CSF. We tested whether hnRNPA2/B1 might also be present in CSF vesicles. CSF EVs were isolated from patients less than 2 years or greater than 70 years of age and subjected to immunoblot (**Figure 5I**). As hypothesized, hnRNPA2/B1 levels were highest in patients less than 2 years and was 81% lower in patients greater than 70 years of age (1.00 SEM ±0.17 vs 0.19±0.09 N = 3,3 p = 0.0138 (**Figure 5J**).

## Discussion

Our results demonstrate temporal fluctuations in CSF EV concentration and miRNA content. EVs were isolated from human CSF samples using a combination of size exclusion to remove potential lipoprotein contaminants and by polymeric precipitation. EVs isolated in this manner contain immuno-reactivity for general markers of EVs. Reductions in EV concentrations were mirrored by reductions in the EV protein ALIX.

The average size of CSF EVs did not change during development based on nanoparticle tracking analysis. Although side populations did appear in patients younger than two years of age, there was not a statistically significant shift in the average size. Nanoparticle tracking analysis has the ability to appropriately assign size frequency distributions to relatively uniform populations. However a recent report has demonstrated that mixed size population analysis by nanoparticle tracking analysis yields broad particle size distributions [27]. This ultimately results in an inability to bin particles into finite size categories with poor multimodal resolution with an inability to often distinguish peak modal values [27] Nevertheless, nanoparticle tracking analysis identified age dependent decreases and this conclusion is supported by the reduction in EV marker proteins.

We also hypothesized that an additional indicator of age may be the content of CSF EVs. Therefore we started our analysis by subjecting CSF to small RNA next generation sequence analysis. The majority of mapped reads aligned to known microRNA genes. INext generation sequencing also identified RNAs not previously reported. Several of these novel EV RNAs are predicted to base pair with the 3′ UTR of mRNAs. For example, we found that Cuff .1222 targeted the 3′ UTR an mRNA with a miTG score of 96.00 using DIANA - microT v3.0. These findings warrant further investigations into whether the novel EV RNAs function as miRNAs.

Microarrays were performed to determine whether miRNAs were differentially expressed in CSF vesicles based on age. While most highly abundant miRNAs were not differentially expressed under the two conditions, hierarchical analysis classified samples correctly into two groups based on age and identified several differentially expressed miRNAs. Further investigations will be required to determine whether differentially expressed miRNAs have an important role in early brain development.

More than 50 highly abundant miRNAs were present in each condition. Bioinformatic analysis of highly abundant miRNAs revealed a consensus recognition sequence which contained overlap with a zip-code motif recognized by hnRNPs [23]. Indeed, we identified hnRNPA2/B1 in CSF vesicles and found age dependent declines in hnRNPA2/B1 isolated from CSF vesicles. The size of hnRNPA2/B1 that we detected is slightly larger than what is predicted. However, the size hnRNPA2/B1 is consistent with differential sumoylation detected for exosome purified hnRNPA2/B1 [23]. We also occasionally found hnRNPA2/B1 immunoreactivity at approximately the predicted size supporting the hypothesis that differential sumoylation may play a role in modulating function within exosomes. Cultures of CPEs did release EVs and also produced EVs that contained hnRNPA2/B1. The significance of hnRNPA2/B1 within CSF vesicles is likely the transport of cellular miRNAs into EVs, although further studies will be needed to address this hypothesis. Our findings that hnRNPA2/B1 may have an important function in CSF EVs are supported by a recent proteomic study has also indicated that many hnRNPs including hnRNPA2/B1 are present in CSF EVs [21]. In conclusion, the temporal dynamics of CSF EV concentrations and miRNA content likely play an important role in coordinating intercellular and intracellular communication in brain development. This study lays the ground work for realizing the full diagnostic and therapeutic potential of EVs and for deciphering their physiologic functions.
